# Relationship between exposure to tumour necrosis factor inhibitor therapy and incidence and severity of myocardial infarction in patients with rheumatoid arthritis

**DOI:** 10.1136/annrheumdis-2016-209784

**Published:** 2017-01-10

**Authors:** Audrey S L Low, Deborah P M Symmons, Mark Lunt, Louise K Mercer, Chris P Gale, Kath D Watson, William G Dixon, Kimme L Hyrich

**Affiliations:** 1 Arthritis Research UK Centre for Epidemiology, Centre for Musculoskeletal Research, Institute of Inflammation and Repair, The University of Manchester, Manchester, UK; 2 NIHR Manchester Musculoskeletal Biomedical Research Unit, Central Manchester NHS Foundation Trust, Manchester Academic Health Science Centre, Manchester, UK; 3 Division of Epidemiology and Biostatistics, Leeds Institute of Cardiovascular and Metabolic Medicine, University of Leeds, Leeds, UK; 4 York Teaching Hospital NHS Foundation Trust, York, UK

**Keywords:** Rheumatoid Arthritis, Epidemiology, Anti-TNF, Cardiovascular Disease

## Abstract

**Objectives:**

Patients with rheumatoid arthritis (RA) are at increased risk of myocardial infarction (MI) compared with subjects without RA, with the increased risk driven potentially by inflammation. Tumour necrosis factor inhibitors (TNFi) may modulate the risk and severity of MI. We compared the risk and severity of MI in patients treated with TNFi with that in those receiving synthetic disease-modifying antirheumatic drugs (sDMARDs).

**Methods:**

This analysis included patients with RA recruited from 2001 to 2009 to the British Society for Rheumatology Biologics Register for Rheumatoid Arthritis starting TNFi (etanercept/infliximab/adalimumab) and a biologic-naïve comparator cohort receiving sDMARD. All patients were followed via physician and patient questionnaires and national death register linkage. Additionally, all patients were linked to the Myocardial Ischaemia National Audit Project, a national registry of hospitalisations for MI. Patients were censored at first verified MI, death, 90 days following TNFi discontinuation, last physician follow-up or 20 April 2010, whichever came first. The risk of first MI was compared between cohorts using COX regression, adjusted with propensity score deciles (PD). MI phenotype and severity were compared using descriptive statistics. 6-month mortality post MI was compared using logistic regression.

**Results:**

252 verified first MIs were analysed: 58 in 3058 patients receiving sDMARD and 194 in 11 200 patients receiving TNFi (median follow-up per person 3.5 years and 5.3 years, respectively). The PD-adjusted HR of MI in TNFi referent to sDMARD was 0.61 (95% CI 0.41 to 0.89). No statistically significant differences in MI severity or mortality were observed between treatment groups.

**Conclusions:**

Patients with RA receiving TNFi had a decreased risk of MI compared with patients with RA receiving sDMARD therapy over the medium term. This might be attributed to a direct action of TNFi on the atherosclerotic process or better overall disease control.

## Introduction

In meta-analyses, patients with rheumatoid arthritis (RA) have a 60% increased risk of myocardial infarction (MI) and a 70% increased risk in mortality from MI compared with the general population.^1^ ^2^ As the development of atherosclerosis in the general population is viewed as an inflammatory process, it is possible that the chronic inflammation associated with RA may accelerate this. Traditional cardiovascular (CV) risk factors do not fully explain the increased risk of MI associated with RA.^3–5^ Drugs inhibiting tumour necrosis factor α (TNFi) have been shown to reduce joint inflammation and associated inflammatory markers; thus, they may also influence the future risk of MI.

The association between TNFi exposure and MI risk has been investigated previously in patients with RA. Some studies found a reduced risk, but others a similar risk compared with treatment with synthetic disease-modifying antirheumatic drugs (sDMARDs).^6–15^ Most of these studies only followed patients for 1–2 years. TNFi may influence the incidence of MI in the short term by stabilising plaque. However, any effect on plaque formation is likely to take much longer. Therefore, the full influence of TNFi on future MI risk may take many years to become apparent.^16^ Also, as MI is a relatively uncommon event, large sample sizes are required to assess this risk.

As well as influencing the occurrence of MI, tumour necrosis factor α (TNFα) may affect the outcome after a CV event. TNFα appears to limit infarct size by preventing or delaying apoptosis of cardiac myocytes and may have a homeostatic role in limiting the amount and duration of damage after an ischaemic insult.^17^ Conversely, neutralising TNFα with antibodies has been shown to reduce infarct size in murine models.^18^ The outcome of MI in patients with RA receiving TNFi therapy has not previously been studied.

We aimed to compare (1) the incidence of MI over the medium term, (2) the severity of MI and (3) the mortality post MI between patients with RA treated with TNFi therapy and those treated with sDMARD therapy.

## Methods

### Study design and setting

The British Society for Rheumatology Biologics Register for Rheumatoid Arthritis (BSRBR-RA) is a UK-wide prospective observational study, established in 2001 to monitor the long-term safety of TNFi and other biological therapies.^19^ UK guidelines restrict the prescription of TNFi in RA to patients with (i) sustained active disease (28-joint disease activity score (DAS28) >5.1 on at least two occasions a month apart) and (ii) who have failed to respond to therapeutic doses of ≥2 sDMARDs (including methotrexate, unless contraindicated) given for ≥6 months.^20^ The TNFi-treated patients included in this analysis were recruited between 2001 and 2005 (etanercept), 2001 and 2008 (infliximab) and 2004 and 2008 (adalimumab). Recruitment to each TNFi drug continued from when that TNFi was licensed in UK until the target of 4000 patients per TNFi was reached. We recruited a comparator cohort of biologic-naïve patients with active disease (guide DAS28>4.2) receiving sDMARD therapies only, between 2002 and 2009. If patients in the comparator cohort were switched to TNFi therapy, they were given the option to re-consent to recruitment to the TNFi cohort (if recruitment to that TNFi cohort was still open); otherwise follow-up was discontinued. Patients could not switch from the TNFi cohort to the sDMARD cohort.

Both cohorts were followed identically via physician and patient questionnaires. Questionnaires were sent to the rheumatology team every 6 months for the first 3 years of follow-up and annually thereafter, regardless of changes to therapy, requesting information on disease details, medication and comorbidities, as well as the occurrence of adverse events. At baseline, physicians were asked if the patient had a history of MI or angina. Patients provided information on hospitalisations via questionnaires every 6 months for the first 3 years of follow-up. For all reports of MI, additional clinical data (discharge summaries, ECG, cardiac enzymes, pathology reports) were requested to aid event verification and classification. All patients were flagged with the Health and Social Care Information Centre for reporting of deaths. Causes of death (COD) were coded from the death certificate using the International Classification of Diseases 10.

In addition, the BSRBR-RA dataset was linked to the Myocardial Ischaemia National Audit Project (MINAP). Established in 1999, MINAP audits the care and outcome of all patients admitted to an acute National Health Service (NHS) hospital in England or Wales with a suspected MI against national standards.^21^ MINAP collects patient-level information including patient demographics, dates of admission, MI phenotype, baseline risk, comorbidities, investigations, details of reperfusion therapy, in-hospital drug treatment, clinical complications and all-cause mortality.

The BSRBR-RA and MINAP datasets were linked using deterministic matching based on surname, forename, gender, unique NHS number, date of birth and postcode. The matching strategy used a maximum of four linkage variables in any one combination. MINAP events which matched to a BSRBR-RA patient were returned to the BSRBR-RA for analysis.

### Participants

**Figure 1:**
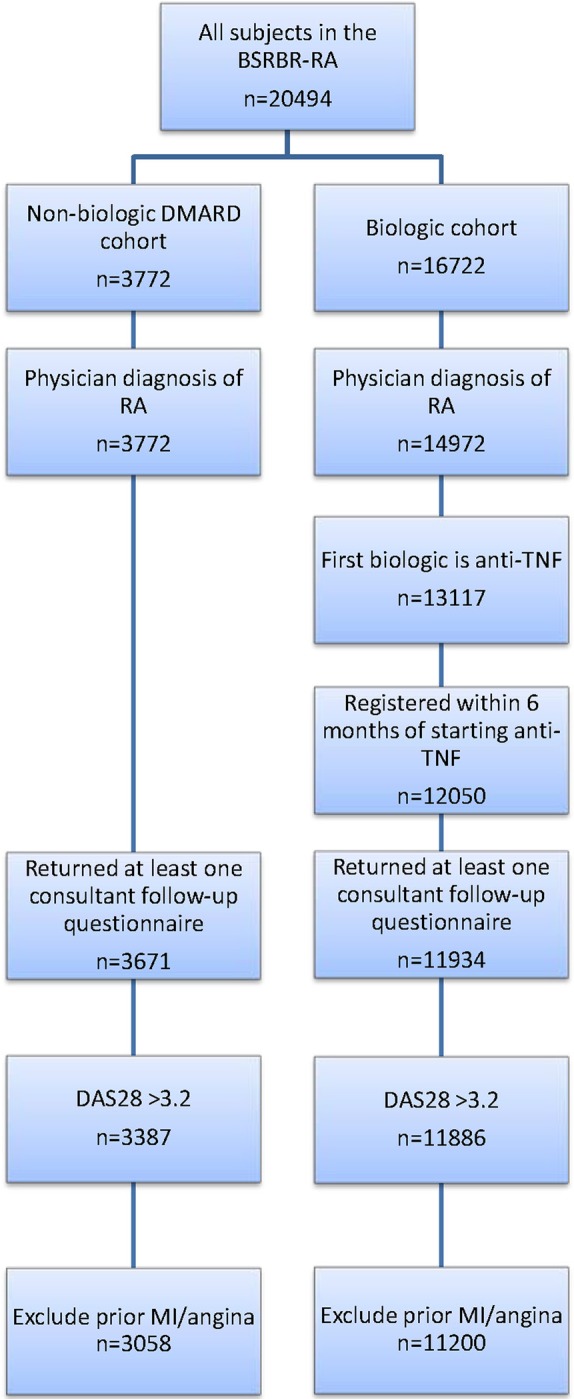
Patient selection for analysis. BSRBR-RA, British Society for Rheumatology Biologics Register for Rheumatoid Arthritis; DAS28, disease activity in 28 joints; DMARD, disease-modifying antirheumatic drug; MI, myocardial infarction; RA, rheumatoid arthritis; TNF, tumour necrosis factor.

This analysis included all patients with a physician diagnosis of RA starting etanercept, infliximab or adalimumab as their first biologic within 6 months of registration with BSRBR-RA, or registered into the sDMARD cohort (figure 1). The analysis was limited to patients with at least moderate disease activity at the start of therapy (DAS28≥3.2) and with no past history of MI or angina. All patients had to have at least one returned rheumatology team follow-up questionnaire to confirm treatment start.

Written consent was obtained from all patients according to the Declaration of Helsinki. Approval for the BSRBR-RA was given by the North West Multicentre Research Ethics Committee (reference no: 00/8/53). The National Institute for Cardiovascular Outcomes Research (NICOR) which includes MINAP (Ref: NIGB: ECC 1-06 (d)/2011) has support under Section 251 of the NHS Act 2006 to use patient information for medical research without requiring additional consent. The data linkage in this analysis was approved by the MINAP Academic Group.

### Verification of MI

All potential MIs reported to BSRBR-RA, MINAP or both were verified using the American Heart Association/European Society of Cardiology (AHA/ESC) criteria for MI.^22^ If a reported event could not be verified against the AHA/ESC criteria for MI, but the patient received thrombolysis or primary angioplasty or died with MI recorded as the underlying COD, this was also considered to be a verified MI.

### Drug exposure models and statistical methods

The primary event was the first verified MI for each patient. Crude incidence rates of first MI with 95% CIs were calculated using a Poisson distribution. For the TNFi cohort, follow-up started on the first day of treatment. For the sDMARD cohort, follow-up started on the date of registration. MIs were attributed to TNFi therapy if they occurred on drug or within 90 days of drug discontinuation. Follow-up was censored at first verified MI, death, date of last physician follow-up or 20 April 2010, whichever came first. The risk of first MI was compared between TNFi-treated and sDMARD-treated patients using a COX proportional hazards model, adjusted for deciles of propensity score (PD). This was presented using HRs with 95% CI. Sensitivity analyses included (1) analysing the data using an ever-exposed drug model (ie, all exposure time following first dose of TNFi) and (2) trimming of the PD at 5%.

Baseline confounders were specified a priori and entered into a logistic regression model to generate a PD, reflecting the likelihood of receiving TNFi depending on covariates. These covariates were age, gender, DAS28, disease duration, health assessment questionnaire score, whether the patient had used ≥4 sDMARDs prior to study registration, whether the patient was recruited to the register before 30 June 2004 (the approximate midpoint of study recruitment, chosen to account for temporal changes in baseline disease severity over time),^23^ hypertension, diabetes, chronic lung disease, smoking (ever/never), antiplatelet therapy, non-steroidal anti-inflammatory drugs (NSAID)/cyclooxygenase inhibitor (COX)-2 inhibitor use, glucocorticoid use and statin use. Missing data were imputed by multiple imputation by chained equations (MICE).^24^ The imputation model included whether the patient experienced an MI, logarithm of the time to MI and the other covariates as described^25^ (see online supplementary web appendix).

10.1136/annrheumdis-2016-209784.supp1 supplementary web appendix

Severity of the MI was defined according to (1) MI phenotype: ST elevation versus non-ST elevation (STEMI vs NSTEMI), (2) presence or absence of cardiac arrest during hospital admission, (3) peak creatine kinase (CK), peak troponin I, peak troponin T and (4) length of hospital stay. These parameters were compared between cohorts using the χ^2^ test for categorical variables and the Wilcoxon rank-sum test for continuous variables. This analysis was limited to those MIs captured in the MINAP registry. For this analysis, patients were divided into three groups based on treatment at the time of their MI: Group 1 (sDMARD) (referent), Group 2 (receiving TNFi therapy at the time of or within 90 days prior to the MI) and Group 3 (exposed to TNFi but outside the 90-day lag window prior to the MI).

Mortality post MI was defined as any death that occurred within 6 months following the initial MI. The results are presented for (1) all MIs irrespective of reporting source and (2) for those MIs with additional data from MINAP. Using logistic regression, the risk of death within 6 months post MI was compared between the three groups, with the sDMARD group as the referent. For all MIs, mortality was adjusted for age and gender. For those MIs with additional data from MINAP, the analysis was adjusted for age, gender and the **M**odified **G**lobal Registry of Acute Coronary Events (MG) score: a composite score including age, heart rate, systolic blood pressure, creatinine, cardiac arrest at admission, ST segment deviation, elevated cardiac enzymes and loop diuretic use at admission.^26^ ^27^ Higher scores indicate a higher probability of death (range: 0–274). Missing data for components of MG were replaced using multiple imputation.^28^ The risk of death was estimated using logistic regression. ORs with 95% CI were presented. All analyses were performed using Stata V.13 (StataCorp, College Station, Texas, USA).

## Results

### Baseline characteristics

A total of 14 258 patients (sDMARD: 3058, TNFi: 11 200) were analysed (table 1 and figure 1). At baseline, the TNFi cohort was younger, comprised of proportionally more females, had longer disease duration and higher disease activity and functional disability compared with the sDMARD cohort. Patients in the TNFi cohort were also more likely to receive glucocorticoids and NSAID/COX-2 inhibitors, but were less likely to be on antiplatelet drugs and statins. Patients in the TNFi cohort also had a lower frequency of smoking, hypertension and diabetes compared with the sDMARD cohort. Median duration of exposure to TNFi was 4.1 years (IQR 2.0, 5.8).

**Table 1:** Baseline characteristics of patients in sDMARD and TNFi cohorts

	sDMARD; n=3058	TNFi; n=11 200
Mean age, years (SD)	59.5 (12.5)	55.6 (12.3)
Female, %	75	78
Median disease duration, years (IQR)	6 (1, 15)	11 (6, 19)
Mean DAS28 (SD)	5.3 (1.1)	6.6 (1.0)
Mean HAQ score (SD)	1.5 (0.7)	2.0 (0.6)
Proportion of patients who received ≥4 sDMARDs prior to study registration, %	21	53
Proportion of patients who received methotrexate prior to study registration, %	82	97
Recruited before 30 June 2004, %	19	51
Hypertension, %	30	28
Diabetes, %	6	5
Chronic lung disease, %	19	13
Current/previous smoker, %	62	59
Glucocorticoid, %	22	44
NSAID/COX-2 inhibitor therapy, %	56	63
Antiplatelet therapy, %	7	5
Statin therapy, %	9	5

COX, cyclooxygenase inhibitor; DAS28, disease activity in 28 joints; HAQ score, health assessment questionnaire score; NSAID, non-steroidal anti-inflammatory drugs; sDMARD, synthetic disease-modifying antirheumatic drug; TNFi, tumour necrosis factor α inhibitor.

### Risk of MI in TNFi-treated patients compared with sDMARD-treated patients

There were 58 verified first MIs during a median of 3.5 years follow-up in the sDMARD cohort (total follow-up 10 337 person-years (pyrs)) and 194 MIs during a median of 5.3 years follow-up in the TNFi cohort (total follow-up 55 636 pyrs) (table 2). The crude incidence rate of first MIs per 10 000 pyrs was 56 (95% CI 46 to 73) in the sDMARD cohort and 35 (95% CI 30 to 40) in the TNFi cohort. The median time to first MI was 1.56 years (IQR 0.89, 3.43) in the sDMARD cohort and 2.43 years (IQR 1.41, 3.96) in the TNFi cohort.

**Table 2:** Risk of MI compared between sDMARD and TNFi cohorts

	sDMARD; n=3058	TNFi; n=11 200
Median duration of follow-up per patient, years (IQR)	3.5 (1.8, 4.9)	5.3 (3.6, 6.4)
Total person-years of exposure, pyrs	10 337	55 636
Primary drug exposure model: on-TNFi+90 days		
Number of verified first MIs	58	194
Crude incidence rate of verified first MI per 10 000 pyrs (95% CI)	56 (43 to 73)	35 (30 to 40)
Unadjusted HR (95% CI)	Referent	0.78 (0.58 to 1.05)
HR adjusted for age and gender (95% CI)		1.19 (0.89 to 1.59)
HR after adjusting for PD* (95% CI)		0.61 (0.41 to 0.89)
Sensitivity analyses		
In subjects ever exposed to TNFi; PD-adjusted HR (95% CI)		0.67 (0.46 to 0.96)
Trimming the PD at 5%; PD-adjusted HR (95% CI)		0.56 (0.34 to 0.93)

*Deciles of propensity score (PD). The PD included age, gender, DAS28, disease duration, health assessment questionnaire score, whether the patients used four or more sDMARDs prior to study registration (yes/no), whether the patients were recruited to the register before or after 30 June 2004, hypertension, diabetes, chronic lung disease, smoking (ever/never), antiplatelet therapy, NSAID/COX-2 inhibitor use, glucocorticoid use and statin use.

COX, cyclooxygenase inhibitor; DAS28, disease activity in 28 joints; MI, myocardial infarction; NSAID, non-steroidal anti-inflammatory drugs; sDMARD, synthetic disease-modifying antirheumatic drug; TNFi, tumour necrosis factor α inhibitor.

Compared with the sDMARD cohort, the unadjusted HR of MI in the TNFi cohort was 0.78 (95% CI 0.58 to 1.05). After adjustment using PD, the risk of MI in the TNFi cohort was 0.61 (95% CI 0.41 to 0.89), indicating a 39% decreased risk of MI, compared with the sDMARD cohort. There were 276 verified MIs in the group who were ever exposed to TNF (ie, when all person-time following first dose of TNFi was included). Adjustment using PD showed a result similar to the primary model: HR 0.67 (95% CI 0.46 to 0.96) (table 2). Similar results were also found when propensity scores were trimmed at 5% (table 2).

### Severity of MI

Of the 334 verified first MIs (sDMARD: 58, ever exposed to TNFi: 276), 136 MIs were captured by BSRBR-RA only, 78 were captured by MINAP only and 120 were captured in both datasets. There were no significant differences (data not shown) in the age, gender or the proportion of patients receiving TNFi therapy in those MIs with MINAP data and those without.

Of the 198 MI patients with MINAP data (59%), 35 were biologic-naïve (Group 1), 108 were receiving TNFi therapy at the time of or within 90 days prior to the MI (Group 2) and 55 had prior exposure to TNFi (Group 3) (table 3). Overall, there were no statistically significant differences in markers of severity between the three groups, although there was a trend towards higher peak CK in patients on TNFi at the time of MI (table 3).

**Table 3:** Severity of MI compared between sDMARD and TNFi cohorts

Number of verified first MIs with additional MINAP data	Group 1 (sDMARD), n=35	Group 2 (on TNFi at the time of or within 90 days prior to MI), n=108	Group 3 (exposure to TNFi more than 90 days prior to MI), n=55	p Value
Proportion of patients with STEMI, n (%)	16 (46)	53 (49)	27 (49)	0.32
Cardiac arrest, n (%)	3 (9)	5 (5)	5 (9)	0.48
Median peak CK, IU/L (IQR)	290 (172, 1598)	691 (150, 1293)	286 (125, 660)	0.19
Median peak troponin I, μg/L (IQR)	5.0 (1.3, 7.2)	7.4 (1.1, 22.8)	7.6 (1.5, 29.0)	0.46
Median peak troponin T, μg/L (IQR)	0.7 (0.3, 2.3)	0.9 (0.2, 2.3)	0.8 (0.2, 2.1)	0.95
Median length of hospital stay, days (IQR)	6 (5, 9)	6 (4, 8)	6 (4, 11)	0.46

CK, creatine kinase; MI, myocardial infarction; MINAP, Myocardial Ischaemia National Audit Project; sDMARD, synthetic disease modifying anti-rheumatic drug; STEMI, ST-elevation myocardial infarction; TNFi, tumour necrosis factor α inhibitor.

### Post-MI mortality

Of the 334 patients with first MIs, 77 (23%) died within 6 months of their MI: 12 (21%) in Group 1, 25 (13%) in Group 2 and 40 (48%) in Group 3 (exposed, but had stopped TNFi >90 days before their MI) (table 4). Compared with the sDMARD-treated group (Group 1), the age and gender adjusted OR of death in Group 2 was 0.68 (95% CI 0.31 to 1.47) and that in Group 3 was 3.07 (95% CI 1.42 to 6.62). The median time between stopping the TNFi and MI occurrence was 1.3 years (IQR 0.5, 2.5). Similar trends in mortality risk were observed in the subset of MIs captured by MINAP, but there were very few events, and precision was low (table 4). The median MG scores were similar between the three groups (108 vs 100 vs 112, respectively). The MG score was a significant univariate predictor of 6-month mortality: OR 1.05 (95% CI 1.02 to 1.07) within these subjects.

**Table 4:** Mortality within the 6-months following MI

	Group 1 (sDMARD)	Group 2 (on TNFi+90 days lag at time of MI)	Group 3 (exposure to TNFi more than 90 days prior to MI)
Total number of verified first MIs identified from BSRBR-RA and/or MINAP	58	194	82
Deaths within 6 months, n (%)	12 (21)	25 (13)	40 (48)
Unadjusted OR (95% CI)	Referent	0.61 (0.28 to 1.31)	2.84 (1.33 to 6.04)
OR adjusted for age and gender (95% CI)		0.68 (0.31 to 1.47)	3.07 (1.42 to 6.62)
Number of verified first MIs with MINAP data (% total verified MIs)	35 (60)	108 (56)	55 (67)
Deaths within 6 months, n (%)	2 (6)	3 (3)	11 (20)
Median MG score (IQR)	108 (81 to 131)	100 (84 to 120)	112 (93 to 129)
Unadjusted OR (95% CI)	Referent	0.47 (0.08 to 2.94)	4.13 (0.86 to 19.89)
OR adjusted for age and gender (95% CI)		0.51 (0.08 to 3.21)	4.07 (0.82 to 20.07)
OR adjusted for MG score (95% CI)		0.47 (0.06 to 3.45)	5.40 (0.93 to 31.18)

BSRBR-RA, British Society for Rheumatology Biologics Register for Rheumatoid Arthritis; MG score, modified Global Registry of Acute Coronary Events score; MI, myocardial infarction; MINAP, Myocardial Ischaemia National Audit Project; sDMARD, synthetic disease modifying anti-rheumatic drug; TNFi, tumour necrosis factor α inhibitor.

## Discussion

Previous reports of the association between TNFi therapy and the risk of MI have only followed patients up to 1–2 years and have not had consistent findings. We examined the association between TNFi therapy and the risk of MI over the medium term (median follow-up, 5 years) and used propensity scores to balance differences across a wide range of measured covariates. A 39% reduction in the risk of MI was observed in patients treated with TNFi compared with those on sDMARD therapy. We also report, for the first time, the relationship between severity of and mortality post MI among patients who have received TNFi therapy.

There is a signal that duration of TNFi exposure is related to a reduction in cardiovascular disease (CVD) risk in patients with RA. Bili *et al*^13^ found that use of TNFi for more than the median of 16 months was associated with lower risk of CV events: relative risk 0.31 (95% CI 0.15 to 0.65). In a Swedish study, 2 years follow-up on TNFi was associated with a 32% reduction in the risk of acute coronary syndrome: HR 0.78 (95% CI 0.61 to 1.01).^29^ There is a biologically plausible explanation for our findings as TNFα plays a key role in the pathogenesis of atherosclerosis.^30^ Inflammation is central in all stages of atherosclerosis, including endothelial function, plaque stabilisation and postinfarct remodelling, and thus inhibition of TNFα may influence accumulation and progression of plaque leading to fewer MIs. TNFi may also affect CVD risk via changes in lipid profile, insulin resistance and diabetes risk.^31^ ^32^

It is possible that our study findings are attributable to suppression of inflammation and disease control in general rather than a TNFα-specific effect. Solomon *et al*^33^ observed that disease activity control was associated with fewer CV events. Therefore, the current treat-to-target strategy to lower disease activity in RA may improve pain and function, and also reduce CV risk either through using sDMARDs or biologics or a combination. In our study, disease activity in the comparator cohort may have been suppressed by sDMARD therapy, but to a lesser extent, than in the TNFi cohort, thereby ‘maintaining’ the already increased background MI risk.

Blockade of TNFα may modify the incidence of MI and influence the severity and mortality post MI via postinfarct remodelling.^17^ ^18^ This relationship has not previously been explored because the relevant data are generally not collected within drug registries. For this study, we linked with a national MI database to gain additional data on the MIs. Due to differences in study design and geographical setting between MINAP and BSRBR-RA, the overlap of events was not 100%. However, there were no systematic differences between those with MINAP data and those without. Overall, there were no differences in MI severity between treatment groups using indirect measures (MI phenotype, in-hospital cardiac arrest, troponin levels and length of hospital stay). We observed a trend towards higher peak CK levels in patients on TNFi at the time of MI. However, data on the precise time from MI symptom onset to measurement of cardiac enzymes in relation to reperfusion treatment were not recorded in MINAP. These parameters may influence our results; thus, this finding should be interpreted with caution.

Mortality post MI in the general population is associated with a number of factors (eg, age, gender, severity of MI, comorbidities). We were able to use data from MINAP to calculate the modified GRACE score and include this in the regression model. However, small numbers of events precluded robust conclusions. We observed a difference in the direction of relative risk between the group of patients receiving TNFi at the time of MI and those with prior exposure to TNFi (50% reduction vs fivefold increase, respectively). In the latter group, the median time between stopping the TNFi and MI was 1.3 years. Most patients in the latter group had discontinued their TNFi following an adverse event (MI was not the adverse event), which may imply higher levels of comorbidity in these patients.

This study has several strengths. The prospective design of the BSRBR-RA, detailed data collection and the size of the study population meant that it was possible to adjust for a large number of potential confounders compared with previous publications. Despite the large range of covariates, we were unable to adjust for unmeasured confounders such as cumulative steroid dose. Confounding by indication is an issue with observational studies. If patients with severe active RA are at increased risk of CVD and are also more likely to receive TNFi, one would have expected an increased MI risk in the TNFi group, but instead a reduced risk was observed. Employment of propensity scores to balance confounders is an emerging technique in the field of pharmacoepidemiology. In this analysis, the use of PD to adjust for known confounders was associated with low levels of expected bias (<5%, see online supplementary web appendix). Linkage with MINAP enabled analysis of MI severity, including adjustment by the modified GRACE score (a risk prediction score for MI-related death). This study, which has looked at the effects of TNFi when added to sDMARD therapy, cannot be used to compare the risk of MI between incident TNFi use and incident sDMARD use.

In conclusion, treatment with TNFi therapy for RA was associated with a reduced risk of MI over the medium term compared with sDMARD therapy. This might be attributed to a direct action of TNFi on the atherosclerotic process or better overall disease control or both. Severity of MI and mortality post MI were not associated with TNFi therapy in our dataset, but warrants further exploration in collaborative analyses across other biologic registers.
