# Supplementary material for: Relationship between exposure to tumour necrosis factor inhibitor therapy and incidence and severity of myocardial infarction in patients with rheumatoid arthritis
Source: Ann Rheum Dis. 2017 Jan 10;76(4):654–60. doi: 10.1136/annrheumdis-2016-209784 (PMC5530342; doi:10.1136/annrheumdis-2016-209784)
Supplement: supplementary web appendix [file annrheumdis-2016-209784supp001.pdf]

Web appendix

Development of Propensity Score

The propensity score used in this analysis was generated using the principles outlined in Caruana E. C, S. et al. *A new weighted balance measure helped to select the variables to be included in a propensity score model.* Journal of Clinical Epidemiology 2015; doi:10.1016/j.jclinepi.2015.04.009. The expected bias is defined as the difference in the outcome due to the imbalance between each confounder, taking into account the strength of the relationship between each confounder and outcome. Using a matrix of the beta coefficients of the estimated effect of each covariate on the outcome, the degree of expected bias is calculated. This value is the sum of the beta coefficients, taking into account the direction of the effect, some of which were in a negative direction, decreasing the estimate and some of which were in a positive direction, increasing the estimate, thus cancelling each other out. If the direction of the individual coefficients were ignored, one would then obtain the absolute value of the expected bias. This is a better measure of the degree of bias in the analysis compared to using the beta coefficients as it shows how different the two cohorts are from each other and the degree of bias in the estimated treatment effect. A maximum bias of 5% in either direction was considered to be an acceptable threshold. Deciles of PS were used in the final regression model for confounder adjustment. There were subjects in both sDMARD and TNFi cohorts at every decile i.e. there was overlap.

Missing data

Missing data were imputed for the following covariates: DAS28 score, disease duration, smoking status and HAQ. This was performed using the `ice` command in Stata. Imputation was performed separately for TNFi and sDMARD cohorts, with 20 cycles of imputations each, resulting in 20 unique datasets. Each dataset was analysed using standard regression modelling and the final estimate

combined using the `mim` command (according to Rubin's rules) in Stata. Stata version 12 was used (StataCorp, College Station, Texas).

The proportion of missing baseline data for the baseline covariates was approximately 1% for both sDMARD and TNFi cohorts with the exception of HAQ score. There was a high proportion of missing data for HAQ; 20% in the sDMARD cohort and 5% in the TNFi cohort. This difference can partly be explained by the different method by which HAQ was collected from the sDMARD subjects (via return of questionnaire by post) whereas the HAQ was completed at the time of clinic assessment for TNFi subjects. For medications such as antiplatelet drugs, statins and NSAID/COX-2 inhibitor therapy, usage was recorded at baseline from the patients' prescription list i.e. the information was not gathered as a binary covariate therefore it was not possible to ascertain missingness. The proportion of subjects with missing cardiac enzyme data (either troponin I or troponin T or creatine kinase) was 11% vs 9% vs 11% for drug groups 1,2 and 3 respectively (drug groups as described in Methods).
